# *Trichinella spiralis*: nurse cell formation with emphasis on analogy to muscle cell repair

**DOI:** 10.1186/1756-3305-1-27

**Published:** 2008-08-19

**Authors:** Zhiliang Wu, Lj Sofronic-Milosavljevic, Isao Nagano, Yuzo Takahashi

**Affiliations:** 1 Department of Parasitology, Gifu University Graduate School of Medicine, Yanagido 1-1, Gifu, 501-1194, Japan; 2 Institute for the Application of Nuclear Energy, Banatska 31b, 11080, Belgrade, Serbia and Montenegro

## Abstract

*Trichinella *infection results in formation of a capsule in infected muscles. The capsule is a residence of the parasite which is composed of the nurse cell and fibrous wall. The process of nurse cell formation is complex and includes infected muscle cell response (de-differentiation, cell cycle re-entry and arrest) and satellite cell responses (activation, proliferation and differentiation). Some events that occur during the nurse cell formation are analogous to those occurring during muscle cell regeneration/repair. This article reviews capsule formation with emphasis on this analogy.

## Introduction

Parasites alter physiology and/or morphology of hosts in order to survive in a new environment. It is remarkable how some parasites make a new architecture in the host tissue, by morphological remodeling. *Trichinella spiralis *is a typical example. Parasites build their own home in the infected muscles. The home is a capsule which is composed of a collagenous wall and cellular components. The wall provides some protection to the parasite and the cellular component that takes care of the parasites in terms of metabolism. Because of its function, the name "nurse cell" has been given to the cellular components. Both the wall and nurse cell are of host, not parasite origin. Some parasitologists prefer the term nurse cell complex or capsule rather than the term cyst, because the term cyst is used for cells of parasite origin.

The capsule is prominent in the infected muscle; even an untrained pathologist will not overlook it during microscopic examination. The question that first comes to mind is how does *Trichinella *alter host cells and construct such unique place for living? Does *Trichinella *have some unknown specific tools?

This has been an enigma in spite of extensive studies. As early as 1966, Maier and Zaiman commented on the similarities between some of the changes which occur during nurse cell formation and those in muscle cell regeneration [1]. Steward and Read [2] presented a detailed comparison of ultrastructural and biochemical changes that occur during the two processes mentioned above and found them to be remarkably similar. They introduced the hypothesis that process of regeneration plays a significant role in the initial development of nurse cell. A series of recent studies provide more evidence to strengthen their ideas that *Trichinella *utilizes a repairing process of muscles cells to construct the capsule. In other words, after injury induced by parasite invasion, muscle cells start going through the process of repair, just like after any trauma. *Trichinella *borrows only the initial part of this repair process to construct its own home.

Despommier [3] has already elegantly reviewed the process of capsule formation with emphasis on nurse cell formation. The present review article deals with the whole process of capsule formation but puts more emphasis on the analogy between nurse cell formation and muscle cell repair.

### The analogy between nurse cell formation and muscle cell repair

There are many similarities between the processes of nurse cell formation after *Trichinella *infection and regeneration of muscle cells after injury.

A skeletal muscle cell is susceptible to injury by direct trauma or indirect causes such as neurological dysfunction or innate genetic defects. Due to its remarkable ability of regeneration, an injured muscle cell initiates a finely orchestrated set of cellular responses, resulting in the regeneration of a well-innervated, fully vascularized and contractile muscle apparatus. The process of regeneration includes four stages, as reviewed by Wozniak *et al*. [4]: 1) satellite cell activation; 2) satellite cell proliferation; 3) differentiation and fusion; and 4) self-renewal of satellite cell.

Invasion by *Trichinella *new born larvae also causes muscle cell damage, which initiates the activation of satellite cells undergoing proliferation and re-differentiation [5,6]. In this case, the muscle cell affected by *Trichinella *infection initiates de-differentiation, cell cycle re-entry and arrest [7-11].

During this process, many events are similar in both nurse cell formation and muscle regeneration, for example, increase in the amount of sarcoplasmic matrix, the size and number of nuclei which migrate to the center of muscle fiber from the periphery, the size of affected myofibers, the number of mitochondria, DNA and RNA content, and increase in free ribosomes and intense proliferation of rough endoplasmic reticulum and smooth sarcoplasmic reticulum [2].

### Muscle development and regeneration: an overview

A brief review on muscle genesis and regeneration process will provide basic information for understanding of the nurse cell formation process.

#### Muscle genesis

Skeletal muscles are derived from mesodermal precursor cells which originate from the somites. During embryonic development, mesodermal precursor cells are specific to myogenic lineage (known as myoblasts). Proliferating myoblasts withdraw from the cell cycle and terminally differentiate to myocytes. Finally, mononucleated myocytes specifically fuse to each other to form a multinucleated syncytium, which eventually matures into muscle fibers [12]. During the course of muscle development, a distinct subpopulation of myoblasts fails to differentiate, but remains associated with the surface of the developing myofiber as quiescent muscle satellite cells [13-15].

#### Muscle repair

The early events following muscle injury are muscle cell necrosis and accumulation of inflammatory cells within the damaged site, which is a process of degeneration. The activated mononuclear cells release factors that provide chemotactic signals to other inflammatory cells [16-18]. Neutrophils are first to come, followed by macrophages which phagocytose cellular debris and affect other aspects of muscle regeneration by activating myogenic cells [19-21].

**Figure 1:**
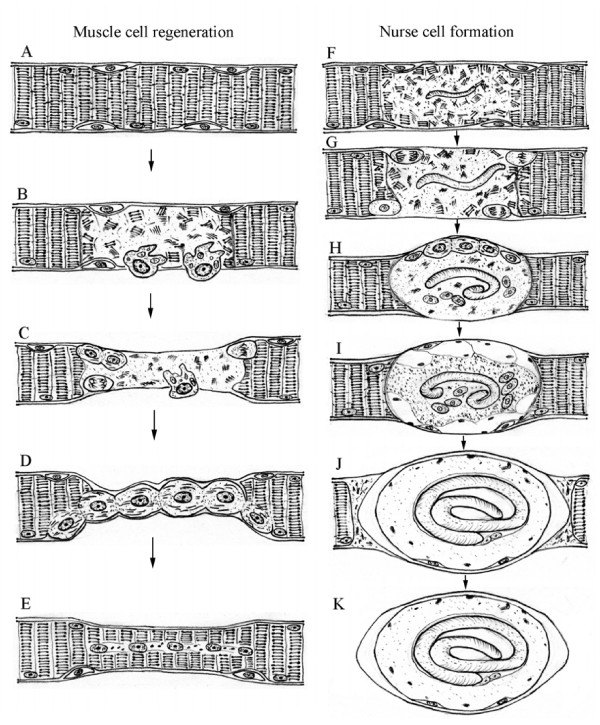
Muscle cell regeneration: A: Normal muscle cell with myonuclei and satellite cells; B: Damaged muscle cell. Muscle injury causes inflammatory response and mononucleated cells are mobilized; C: Necrosis occurs in the damaged site. Macrophages invade the damaged tissue for cleaning up cellular debris. Satellite cells are activated; D: Activated satellite cells proliferate, differentiate and fuse to each other or with existing damaged muscle fibers; E: The regenerated new muscle cell in smaller caliber with centrally-located myonuclei and renewed satellite cells. The figure is modified from the textbook of MYOLOGY by Engel and Franzini-Armstrong. Nurse cell formation: F: Invasion of *Trichinella *larva causes dissolution and complete loss of myofibrillar organization; G: Satellite cells are activated. Basophilic transformation occurs in the infected muscle cell. A septum is formed to limit damaged area; H: Activated satellite cells proliferate, differentiate and fuse to each other or with the infected muscle cell, which provides eosinophilic cytoplasm. The infected muscle cell dedifferentiates, reenters cell cycle and arrests at G2/M. There are many hypertrophy nuclei; I and J: The eosinophilic cytoplasm (which is provided by satellite cells) increases in volume and the basophilic cytoplasm (which originates from infected muscle cell) decreases in volume; K: The mature nurse cell is formed. The cytoplasm of nurse cell is eosinophilic.

Following muscle degeneration, the repair process of muscle is activated. The activation and proliferation of satellite cells are important events necessary for muscle regeneration. The proliferation of satellite cells provides a sufficient source of new myonuclei for muscle repair. Satellite cells differentiate and fuse to each other or with existing damaged fibers for repair to form new myofibers [4,22]. The fundamental morphological characteristics are that newly formed myofibers have small caliber with centrally located myonuclei (Fig 1).

### Capsule formation

Capsule formation (also known as cystogenesis) has been extensively studied by many authors. It involves complex steps and events which take place over a 20 day period from the time of initial larval invasion to the completion of the nurse cell [3].

Infection causes profound changes in host muscle cells, some of which are, in the beginning, similar with those involved in muscle regeneration. After new born larva invasion, dissolution and complete loss of myofibrillar organization occur [23]. A septum is formed to segregate the affected area (basophilic cytoplasm) from the intact area of the same muscle cell [5]. Infection causes the activation, proliferation and differentiation of satellite cells, which develop into eosinophilic cytoplasm [5,6].

Recent molecular biological studies have shown that many genes and signaling pathways are mobilized in nurse cell formation, for example, mitochondrial pathway mediated and death receptor pathway mediated apoptosis signaling, TGF-β signaling pathway, as well as the genes related to cell differentiation, proliferation, cell cycle control, and apoptosis [6,9-11,24-27]. The terminally differentiated muscle cells re-enter the cell cycle and then arrest in apparent G2/M [7,8,23]. The developed nurse cell contains as many as 100 greatly enlarged nuclei with well developed nucleoli [28] and is surrounded by a collagenous capsule wall and circulatory rete [29,30].

In the following paragraphs, each step of capsule formation is reviewed in detail in comparison with muscle cell regeneration.

#### Dynamic changes in infected muscle cell cytoplasm

The muscle cell will disintegrate if the damage is so extensive that cell can not be repaired, which is known as necrosis. This dead area is removed by scavenger cells such as macrophages through the phagocytosis process. When the damage is light, the muscle cell may undergo apoptosis or recover from the damage by repairing itself. In the case of *Trichinella *infection, the invasion itself does not seem to cause severe damage to the muscle cell. As such, the infected muscle cell does not undergo necrosis but it undergoes apoptosis instead. In the following paragraph, recent progress about the fate of infected and damaged muscle cells is discussed, because such information seems to be indispensable to better understand the process of nurse cell formation.

First of all, during the process of nurse cell formation, the existence of two kinds of cytoplasm within the nurse cell should be recognized, basophilic and eosinophilic cytoplasm [5]. Basophilic cytoplasm is formed by the transformation of the infected muscle cell after newborn-larva invasion ("basophilic transformation") [31,32]. The eosinophilic cytoplasm is derived from satellite cells and joins the nurse cell (This is discussed below). In the beginning of nurse cell formation, the basophilic cytoplasm is dominant, and as the nurse cell formation proceeds, the basophilic cytoplasm decreases in size and the eosinophilic cytoplasm increases in size with the ratio changing in a reciprocal manner. Consequently, the basophilic cytoplasm disappears from the nurse cell (Fig 1).

As for the basophilic cytoplasm, morphological and molecular biological data are available. The initial changes include disintegration of sarcomeres, lysis of myofilaments, increases in the amounts of rough and smooth endoplasmic reticulum, and hypertrophy of nuclei [31,32]. Morphological signs are identified as apoptosis [5,24]. There are irregular shaped nuclei with scattered and dense heterochromatin in basophilic cytoplasm. The mitochondria swelled and disappeared in the early phase of infection, and were replaced by new mitochondria that were smaller in size than those in normal muscle cells and had a hyper-density matrix, which was in good agreement with features of mitochondrial pyknosis in apoptosis [33,34]. TUNEL assay indicated that there was DNA fragmentation in some of the enlarged nuclei [27].

**Figure 2:**
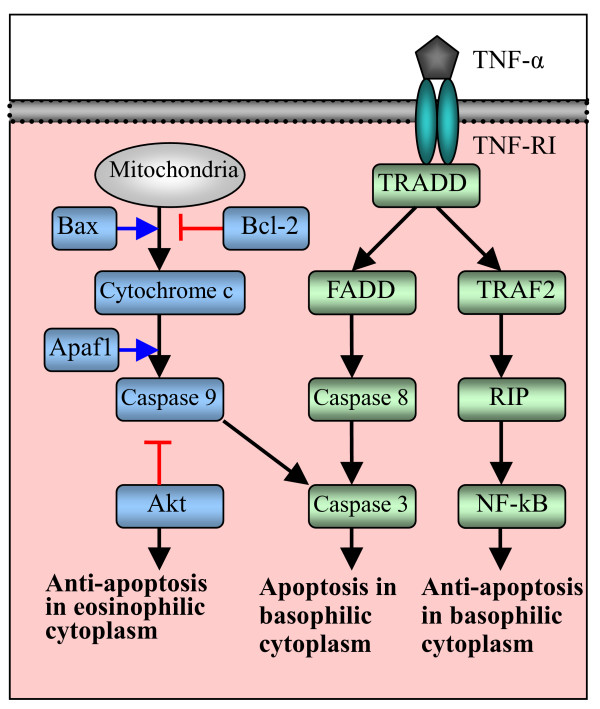
Schematic illustration of the involvement of death receptor pathway (right half) and mitochondrial pathway mediated (left half) apoptosis in nurse cell formation. Upon binding with TNF-α, TNF-RI recruits TRADD which functions as a platform adapter that recruits several signaling molecules. The recruitment of TRADD and FADD results in autocatalytic activation of procaspase 8. Activated caspase 8 cleaves effector procaspase 3 which plays a role in apoptosis in the basophilic cytoplasm of *Trichinella *infected muscle cells. On the other hand, the binding of TNF-α and TNF-RI induces the sequential recruitment of TRADD, TRAF2 and RIP, which leads to the activation of NF-kB. The activated NF-kB acts for anti-apoptosis in the basophilic cytoplasm. In mitochondrial pathway, Bax induces apoptosis by forming the membrane pore in mitochondria from which cytochrome c is released. Cytochrome c activates caspase 9 which in turn activates caspase 3 to induce apoptosis in infected muscle cells. As a co-factor, Apaf-1 plays a role with caspase 9 in apoptosis in the basophilic cytoplasm. On the other hand, Akt plays an anti-apoptosis role in the eosinophilic cytoplasm by inactivating proapoptotic proteins such as Bad and caspase 9. This figure referred the review by Gupta [35].

More light on the mechanisms of apoptosis in the basophilic cytoplasm has been thrown by molecular experiments, which showed that many apoptosis-related genes were involved (Fig 2 and Table 1). There are two principal pathways for apoptosis initiation, the mitochondrial pathway and the death receptor pathway [35]. The up-regulated expressions of mitochondrial pathway mediated apoptosis factors (Bcl-2 associated protein X: BAX, Apoptotic protease activating factor 1: Apaf-1 and caspase 9) and death receptor pathway mediated apoptosis factors (tumor necrosis factor-alpha: TNF-α, TNF receptor I, TNF receptor-associated death-damain: TRADD, caspase 8 and caspase 3) were observed in the basophilic cytoplasm of infected muscle cells, suggesting that both signaling pathways are activated in the cytoplasm (Fig 2) [24-27].

**Table 1:** Expression change of the genes related to apoptosis after *Trichinella *infection

Gene Name	Description	Expression change	
		
		Ts	Tp ^a^
tumor necrosis factor receptor 1 (TNFR1)	TNF-medicated apoptosis	↑	↑
proline dehydrogenase (oxidase) 2 (Prodh2)	mitochondria-mediated apoptosis	↑	↑
Bcl2-interacting killer-like (Biklk)	Bcl family protein; induction of apoptosis	↑	↑
B-cell leukemia/lymphoma 6 (Bcl6)	apoptosis; caspase activation	↑	↑
programmed cell death protein 11 (Pdcd11)	hydrolase activity; apoptosis	↑	↑
clusterin (CLU)	anti- or proapoptotic activity	↑	↑
nuclear protein 1 (Nupr1)	induction of apoptosis; response to stress	↑	NC ^b^
p53	apoptosis, DNA repair, cell cycle arrest	↑	↑
p21	apoptosis, cell cycle arrest	↑	↑
MDM2	apoptosis, negative regulator of p53	↑	↑
Bcl-2 associated protein X (BAX)	mitochondria-medicated apoptosis	↑	↑
Apoptotic protease activating factor 1 (Apaf1)	mitochondria-medicated apoptosis	↑	↑
Caspase 9	mitochondria-medicated apoptosis	↑	↑
protein kinase B (PKB)	promote cell survival and prevent apoptosis	↑	↑
tumor necrosis factor-alpha (TNF)	cell proliferation, differentiation, apoptosis	↑	UR ^c^
TNFR1-associated via death domain (TRADD)	adaptor of TNFR1 mediated apoptosis	↑	UR
Caspase 8	apoptosis	↑	UR
Caspase 3	apoptosis	↑	UR
TNF receptor-associated factor 2 (Traf2)	mediator of anti-apoptotic in TNFR1 signal	↑	UR
Receptor interactive protein (RIP)	mediator of anti-apoptotic in TNFR1 signal	↑	UR

a: Ts: *Trichinella spiralis*; Tp: *T. pseudospiralis*

b: NC: no change

c: UR: no result

The fate of basophilic cytoplasm is thus clear; it disappears through the process of apoptosis in spite of the up-regulation of the genes for anti-apoptosis (TNF receptor associated factor 2: TRAF2, and receptor interactive protein: RIP). In fact, acid phosphatase activity was found to be high in basophilic cytoplasm, suggesting the presence of destructive processes [36]. On the other hand, the eosinophilic cytoplasm seems to tell a different story. This cytoplasm seems to be metabolically active, engaging in some metabolic transportation, because alkaline phosphatase activity, not acid phosphatase activity, was detected in the eosinophilic cytoplasm [36].

The eosinophilic cytoplasm is also exposed to stress from the parasite, and apoptosis genes are up-regulated. Interestingly, however, anti-apoptosis genes are also up-regulated [24-26]. Thus, the eosinophilic cytoplasm is characterized by co-existence of apoptotic and anti-apoptotic mechanisms and retains its activity as a result of balance between apoptosis and anti-apoptosis.

cDNA microarray analysis indicated that some other genes may be involved in the apoptosis occurred in infected muscle cells, for example, Bcl6, clusterin (CLU), Bcl2-interacting killer-like (Biklk), programmed cell death protein 11 (Pdcd11), proline dehydrogenase 1 (Prodh1) and Prodh2 [11]. These genes function in inducing apoptosis or prevent apoptosis, and are related with cell growth and survival [37-46]. The up-regulated expressions of these genes suggest that they engage in the apoptosis and anti-apoptosis in infected muscle cell through different mechanisms.

#### Satellite cell activation, proliferation and differentiation

Each myofiber is surrounded by a single sheet (basal lamina). Within this sheet, there is another cell, the satellite cell. As mentioned in the previous paragraph, satellite cells are myoblasts which differentiate to a new muscle cell when the muscle is injured. Muscle damage triggers such activation and proliferation of satellite cells. Thus, the satellite cells can continuously supply the new muscle cells even if the muscle is damaged. Some of these events are common with the myopathy provoked by *Trichinella *infection.

##### 1. Satellite cell in muscle regeneration

Activation of muscle satellite cells appears to be an important step in the ability of muscle to regenerate. In the course of muscle regeneration, satellite cells first exit their normal quiescent state to start proliferating. After several rounds of proliferation, a majority of the satellite cells differentiate and fuse to form new myofibers or to repair damaged ones [22,47]. The process of satellite cell activation and differentiation during muscle regeneration is reminiscent of embryonic muscle development. In particular, the critical roles of the myogenic regulatory factors (MRFs: MyoD, myogenin, Myf5 and MRF4) and paired box genes (Pax 3 and Pax 7) are observed in both processes [48-50].

At the molecular level, activation of satellite cells is characterized by the rapid up-regulation of two MRFs, Myf5 and MyoD. Following muscle injury, MyoD and Myf5 up-regulation appears early, and the activation of expression has been observed in various *in vivo *models for muscle regeneration and in various types of muscle [15,51-55]. MRF4 likely plays a role in maturation of regenerated myofibers. After the satellite cell proliferation phase, myogenin and MRF4 are up-regulated in cells beginning their terminal differentiation program. This is followed by the activation of cell cycle arrest protein p21 (cyclin-dependent kinase inhibitor 1A) and permanent exit from the cell cycle. The differentiation program is then completed with the activation of muscle specific proteins, such as MGC, and fusion to damaged muscle cells [56-59].

##### 2. Satellite cell in nurse cell formation

Activation and proliferation of satellite cells occur in *Trichinella *infected muscles. A linear alignment of satellite cell nuclei is observed in the periphery of infected cells along their long axis of myofibers [5]. Myogenic regulatory factors (MyoD and myogenin) were over-expressed in infected muscle tissue of both *T. spiralis *and *T. pseudospiralis *infection, and the MyoD factor is highly expressed in the satellite cells of infected muscle cells [6].

cDNA microarray analysis has revealed that several other genes important for differentiation of satellite cells are up-regulated during nurse cell development, as shown in Table 2, including Pax7, desmin, M-cadherin, Numb, manic fringe homolog (Mfng), Deltex 1 (Dtx1), myocyte-specific enhancer factor 2C (MEF2), pre B-cell leukemia transcription factor 1 (Pbx1), and nuclear factor of activated T cells (NFAT) [9-11].

**Table 2:** Expression change of the genes related to muscle development, myogenesis and regeneration after *Trichinella *infection

Gene Name	Description	Expression change	
		
		Ts	Tp ^a^
MyoD	skeletal muscle development and differentiation	↑	↑
myogenin	skeletal muscle development and differentiation	↑	↑
galectin 3	skeletal muscle development	↑	↑
Casitas B-lineage lymphoma (CBL)	suppressing transformation; muscle degeneration	↑	↑
manic fringe homolog (Drosophila) (Mfng)	promoting differentiation by repression of Notch signaling	↑	↑
eyes absent 2 homolog (Drosophila) (Eya2)	muscle development; myogenesis	↑	↑
ski proto-oncogene; (c-ski)	cell differentiation and transformation	↑	↑
insulin-like growth factor binding protein 4 (Igfbp4)	skeletal muscle development	↑	↑
galectin 1	myoblast differentiation and fusion; myotube growth	↑	↑
dickkopf homolog 4 (Dkk4)	limb development	↑	↑
bone morphogenetic protein 4 (Bmp4)	skeletal development; angiogenesis	↑	↑
T-box 15 (Tbx15)	limb development of limb	↑	↑
pre B-cell leukemia transcription factor 1 (Pbx1)	embryonic development and differentiation;	↑	↑
numb gene homolog (Drosophila) (Numb)	cell proliferation and differentiation in muscle development	↑	↑
paired box gene 7 (Pax7)	development; organogenesis; cell differentiation	↑	↑
myocyte enhancer factor 2C (MEF2C)	regulation of transcription; myogenic differentiation	↑	↑
nuclear factor of activated T cells (NFAT)	transcriptional activator activity; cytokine production	↑	↑
deltex 1 homolog (Drosophila) (Dtx1)	myogenesis, muscle development and proliferation	↑	↑
desmin	cytoskeleton organization; muscle contraction	↑	↑
homeo box, msh-like 1 (Msx1)	organ morphogenesis; skeletal development	↑	↑
myeloid leukemia factor 1 (MLF1)	cell differentiation; development; hemopoiesis	↓	↓
chordin-like 2 (Chrdl2)	skeletal development	↑	NC ^b^
paired box gene 3 (Pax3)	cell migration and proliferation; muscle development	↑	NC
Transforming growth factor 2	controls proliferation, differentiation and transformation	↑	↑
smad 2	Transducer of TGF signal pathway, cell proliferation and differentiation	↑	NR ^c^
smad 4	Transducer of TGF signal pathway, cell proliferation and differentiation	↑	NR

a: Ts: *Trichinella spiralis*; Tp: *T. pseudospiralis*

b: NC: no change

c: UR: no result

Pax7 and desmin are specifically expressed in quiescent and activated muscle satellite cells and have been used as a molecular marker of muscle satellite cell [60,61]. The over-expression of Pax7 and desmin indicate that the satellite cells in infected muscle were activated and in proliferating.

M-cadherin, a marker of satellite cells and expressed at the cell surface of proliferating satellite cells, is highly expressed during prenatal development in myogenic cells of somatic origin, in myoblasts forming small muscle bundles in developing limb bud, in myoblasts, and in regenerating skeletal muscle [62,63]. An over-expression of M-cadherin was observed in *T. pseudospiralis*, but not in *T. spiralis*, thus suggesting the differential expression may play a role in the pathology induced by *T. pseudospiralis *by regulating the satellite cells of infected muscle cells.

Multiple mechanisms may involve in the regulation of differentiation initialed by *Trichinella *infection. One of those is the Notch signal pathway. Notch signaling plays an important role in tissue morphogenesis both during development and during postnatal regeneration of skeletal muscle [64]. Numb, Mfng and Dtx1, the regulators of the Notch signaling pathway [64-67], were up-regulated in both *T. spiralis and T. pseudospiralis *infected muscle tissues, suggesting that this signaling pathway is likely to be involved in the activation and differentiation of satellite cells or infected muscle cells.

The factor MEF2 is involved in the activation of muscle-specific gene expression, and acts in concert with MRFs in muscle cell differentiation [12,68]. The factor NFAT plays a role in regulation of MRFs expression in satellite cells [69].

The factor MRF4 behaves differently. During muscle cell regeneration, MRF4 plays a role in the maturation of regenerated myofiber [58,68]. After trauma there is an up-regulation of MRF4 after initiating a terminal differentiation program. In *Trichinella *infection, no expression change of MRF4 was observed during the nurse cell formation [6]. This difference may reflect the fact that the satellite cell cannot be "matured" as a new muscle cell, but instead de-differentiates to the nurse cell.

#### Roles of insulin-like growth factor (IGF) in satellite cell activation and differentiation

The IGF I signaling pathway in muscle biology has been an interesting issue as a result of the fact that IGF I induces both proliferation and differentiation via the type I receptor [70]. As a key factor, IGF-I involves proliferation and differentiation of satellite cells during muscle regeneration [71-73]. There is over-expression of IGFs, for example, IGF I, IGF I receptor, IGF binding protein 2 (IGFBP2), IGFBP4 and IGFBP5 [9,11], in *Trichinella *infected muscle tissue, which suggests that these factors likely play an important role in nurse cell formation.

The binding of IGF-I to the IGF-I receptor induces phosphorylation of the receptor, which then mainly function at 3 different levels.

**Figure 3:**
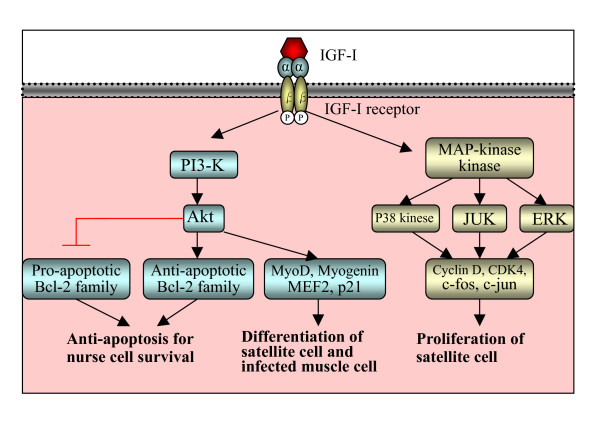
Schematic illustration of IGF-I signaling pathway in nurse cell formation. The binding of IGF-I to IGF-I receptor induces phosphorylation of the receptor, which acts through MAP-kinase kinase and/or PI3-K. Via the MAP kinase pathway, it activates cell cycle progression genes (cyclin D, cdc4, *c-fos *and *c-jun*) which proliferates satellite cells after *Trichinella *infection. Via the PI3-K/Akt pathway, it modulates the expression of muscle differentiation genes (p21, MyoD, Mef-2 and myogenin) which involve in the redifferentiation of satellite cells and differentiation of infected muscle cells. Also the activation of PI3-K/Akt inhibits proapoptosis by Bcl-2 family (Bax, Bad) and induces anti-apoptotic function by Bcl-2 family (Bcl-X), which contributes to the survival of nurse cells. This figure referred the review by Mourkioti and Rosenthal [70].

First, IGF-I has been shown to activate myoblast proliferation through the mitogen activated protein kinase (MAP kinase) signaling pathway, which activates cell cycle progression markers, such as cyclin D, cyclin-dependent kinase 4 (CDK4), c-fos, c-jun [74-76]. It was found that in *Trichinella *infected muscle, expression of IGF-I, IGF-I receptor, IGFBPs, MAP kinase kinase, cyclin D2, cyclin D3, CDK4 and *c-jun *were up-regulated [9,11], suggesting that IGF-I likely plays role in the proliferation of satellite cells and cell cycle reentry of infected muscle cell during nurse cell formation through MAP kinase signaling (Fig 3).

Secondly, IGF induces differentiation of myoblast via the phosphatidylinositol 3-kinase (PI3-K) pathway, which activates Akt and subsequently modulates expression of terminal muscle differentiation markers, such as p21, MyoD, myogenin and MEF2 [74,75,77]. In *Trichinella *infection, expression of Akt, MyoD, myogenin and p21 was greatly increased during 13–28 dpi [6,24-26]. Immunostaining indicated that increased expression of Akt is limited in the eosinophilic cytoplasm which originates from satellite cells [24], and MyoD is limited in the satellite cells of infected muscle cells [6]. The cDNA Microarray analysis showed that the expression of MEF2 was up-regulated in the *T. spiralis *infected muscle tissues [9,11]. Therefore, through the PI3-K-Akt signaling pathway, IGF-I is likely to play a role in the differentiation of activated satellite cell after *Trichinella *infection (Fig 3).

Thirdly, through the PI3-K/Akt signaling pathway, IGF-I also has an effect on cell survival by inhibiting proapoptotic proteins of Bcl-2 family (Bax and Bad), and by inducing anti-apoptotic proteins of Bcl-2 family (Bcl-X) [78,79]. In *Trichinella *infection, there was an increased expression of Bax protein in the basophilic cytoplasm of infected muscle cell at an early stage of infection (18 dpi), but the expression decreased to an undetectable level at a late stage of infection (48 dpi) [24]. Kinetics of this gene expression corresponded to the process of nurse cell formation [24-26]. Therefore, IGF-I might be involved in modulating apoptosis and anti-apoptosis, leading to the survival of infected muscle cells (Fig 3).

#### Factors for cell cycle reentry and arrest

Following invasion of new born larvae, the infected muscle cell withdraws from the G0 cell cycle and re-enters the cell cycle [7]. The enlarged nuclei possess an approximate 4N complement of DNA. The increased DNA synthesis is completed by 5 dpi, and then is suspended throughout the course of infection, which indicated that cell cycle is arrested at G2/M. The molecular mechanism of cell cycle reentry and arrest during infection remains unclear, but recent studies have provided further insight.

The phenomenon of cell cycle arrest may be unique to the nurse cell formation because no comparative phenomena were reported in muscle genesis or muscle repair processes, so far as the present authors are aware.

##### 1. The genes related to regulation of cell cycle in nurse cell formation

As shown in Table 3, expression change of many cell cycle-related factors was observed in *Trichinella *infected muscle tissue, for example, retinoblastoma (Rb), CDK4, cyclin C, cyclin B2, cyclin D2 and cyclin D3, CLU, G0/G1 switch gene 2 (G0S2), inhibitor of DNA binding 2 (Id2), myeloblastosis oncogene (Myb), and N-myc downstream regulated gene 2 (Ndrg2) [9,11]. These factors have already been elaborated by other authors. For example, different cyclins bind specifically to different CDKs to form distinct complexes at specific phases of the cell cycle and thereby drive the cell from one stage of the cycle to another [80,81]. Upon stimulation, D-type cyclins assemble CDK4 and CDK6 to form complexes, which facilitate cells to exit from the G0 phase and re-enter the cell cycle of G1 cell cycle phase [82-84]. Therefore, increased expression of cyclin D2, cyclin D3 and CDK4 is probably involved in the cell cycle reentry after infection.

**Table 3:** Expression change of the genes related to cell cycle regulation after *Trichinella *infection

Gene Name	Description	Expression change	
		
		Ts	Tp ^a^
retinoblastoma 1 (Rb1)	negative regulation of cell growth and progression via cell cycle	↑	↑
ring-box 1 (Rbx1)	cell cycle regulation of G1/S transition	↑	↑
cyclin-dependent kinase inhibitor 1A (P21)	cell cycle arrest; negative regulation of cell proliferation	↑	↑
cyclin-dependent kinase 4 (CDK4)	cell cycle; cell proliferation; G1/S transition	↑	↑
G0/G1 switch gene 2 (G0s2)	regulation of progression through cell cycle	↓	↓
Granulin	Mitogen, cell cycle progression, cell motility	↑	NC ^a^
cyclin A2	G1/S and G2/M transitions, regulator of CDC2 or CDK2 kinases	↑	NC
cyclin C	regulation of cell cycle	↑	NC
Cyclin D3	cell cycle G1/S transition, regulator of CDK4 or CDK6	↑	UR ^a^
Cyclin D2	cell cycle G1/S transition, regulator of CDK4 or CDK6	↑	UR
Cyclin B2	Cell cycle regulation, TGF beta-mediated cell cycle control	↑	UR
cyclin E1	G1/S transitions, regulator of CDC2	↑	UR
myeloblastosis oncogene (Myb)	regulation of cell cycle; G1/S transition of mitotic cell cycle	NC	↑
CDC20	regulation of cell cycle	↑	UR
cyclin-dependent kinase inhibitor 1B (P27)	controls cell cycle progression at G1, prevents activation of cyclin E-CDK2 or cyclin D-CDK4 complexes	↑	UR
Cullin 3 (Cul3)	Cell cycle arrest, G1/S transition of cell	NC	↓
Cell division cycle 5 (Cdc5)	positive regulator of cell cycle G2/M progression	NC	↓

a: Ts: *Trichinella spiralis*; Tp: *T. pseudospiralis*

b: NC: no change

c: UR: no result

On the other hand, up-regulated expression of retinoblastoma (Rb), p21, p27 (cyclin-dependent kinase inhibitor 1B) and p57 (cyclin-dependent kinase inhibitor 1C) may be responsible for the cell cycle arrest of infected muscle cell [9,26]. These kinds of factors are known to play an important role in the growth arrest of differentiating cells, because they specifically inhibit CDKs, which leads to the withdrawal of cells from the cycle and differentiation [85-87].

As a cyclin-dependent kinase inhibitor, p21 is a critical factor in cell cycle arrest at G2/M [88]. Cells deficient in p21 are unable to maintain stability of the cycle arrest [89]. Introduction of non-functional p21 or a p21 antisense oligonucleotide diminished the G2/M arrest phenotype in cells [90,91]. In *Trichinella *infection, expression of p21 was up-regulated, which increased from 13 dpi, reached a peak at 18 dpi and then decreased at late stage of infection [25,26]. Therefore, p21 is an important factor in cell cycle arrest during nurse cell formation.

The expression changes of several other cell cycle-related genes were also observed in *Trichinella *infection, for example, CLU and G0S2. The expression of CLU was up-regulated, while the expression of G0S2 was down-regulated [11]. It is known that, both genes play roles in regulating the cell cycle. An over-expression of CLU resulted in an increased accumulation of cells at the G0/G1 phases of the cell cycles, accompanied by slow down of cell cycle progression and a reduction of DNA synthesis [92]. High level of CLU causes cell cycle arrest [93,94]. G0S2 is transiently induced upon re-entry of cells into the G1 phase of the cell cycle [95,96]. Therefore, UCL and G0S2 may be involved in the arrest of infected muscle cells.

##### 2. Involvement of TGF-β signaling pathway in cell cyclearrest

**Figure 4:**
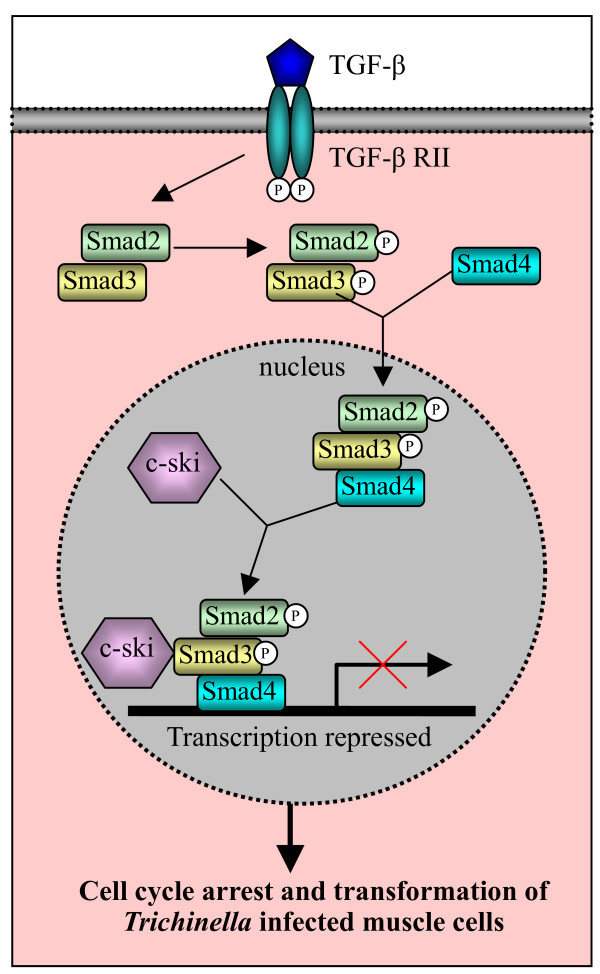
Schematic illustration of the involvement of c-Ski and TGF-β signaling pathway in nurse cell formation. Binding of TGF-β by the type II receptor on the cell surface initiates a cascade of signaling events. Activated type I receptor phosphorylates Smad2 and Smad3 in the cytoplasm, which forms a complex with Smad4. The Smad2/3/4 complex moves to the nucleus and functionally collaborates with distinct transcription factors to turn on or off transcription of many TGF-β-responsive genes. C-Ski acts as a co-repressor to turn off the transcription, which results in the cell cycle arrest and transformation of *Trichinella *infected muscle cells. This figure referred the review by Shi and Massague [97].

One of the important signaling pathways involved in cell cycle arrest is the TGF-β (transforming growth factor) signaling pathway. TGF-β is a ubiquitous cytokine that regulates cell differentiation, proliferation, apoptosis and morphogenesis [97]. Through a series of Smad proteins (Smad 2, Smad 3 and Smad 4), the TGF-β signaling pathway causes cells to cease proliferation and to down-regulates the genes which promote cell cycle progression though the S phase, leading to the arrest of the cell cycle (Fig 4).

Recent studies indicated that the expression of the TGF-β signaling pathway factor genes (TGF-β, Smad2 and Smad4) and *c-ski*, the repressor of the signal pathway, were up-regulated in *Trichinella *infected muscle cells [9-11]. The analysis of expression kinetics showed that the expression of these genes increased at 13 dpi, reached a peak at 23 dpi and then decreased, which is corresponding to the process of nurse cell development. Immunohistochemical analysis indicated that in the early stages of infection, the increased expression of the c-Ski protein was limited to the eosinophilic cytoplasm, while at a later stage of infection the c-Ski protein was limited to the enlarged nuclei in the basophilic cytoplasm, rather than the eosinophilic cytoplasm [10]. These findings provide evidence that the TGF-β signaling pathway is involved in the cell cycle arrest and transformation of infected muscle cells.

#### De-differentiation of infected muscle cell and origin of hypertrophy nuclei

The invasion of new born larvae induces the de-differentiation of infected muscle cell, with features of loss of muscle cell characteristics, change in muscle gene expression and up-regulated expression of cell differentiation related genes (such as MyoD, myogenin, MEF2, Pbx1, Numb, Pax7, Msx and NFAT) in infected muscle tissues [7,9,98-100]. Upon stimulation of larva invasion, infected muscle cells withdraw from the G0 cell cycle and re-enter the cell cycle.

It is commonly thought that newly regenerated fibers are produced by the fusion of activated satellite cells during muscle regeneration. Studies, however, indicated that terminally differentiated myotubes can de-differentiate and Msx genes can be one of the factors to contribute to this process [101-105]. The early event in the de-differentiation of the infected muscle cell may follow the mechanism of de-differentiation in muscle cell regeneration, which was characterized by similar phenomena, for example, the losing of myofibrillar structure, enlarged nuclei and cell cycle re-entry [7,28,98,106-108]. The up-regulated expression of Msx1 and Msx2 in infected muscle tissue supports the proposal that de-differentiation of infected muscle provides hypertrophic nuclei [9,11].

cDNA microarray analysis showed that some other genes may be involved in the de-differentiation of infected muscle cells, for example, galectin 1 and galectin 3, Nanog [11]. The expression of galectin 1 and galectin 3 were up-regulated in *Trichinella *infection. Both genes induce a non-committed myogenic cell within the dermis to expression myogenic markers, increases the terminal differentiation of committed myogenic cells and play a role in skeletal muscle determination, differentiation and regeneration [109-111], suggesting their potential involvement in the de-differentiation of infected muscle cells.

There are as many as 100 hypertrophic nuclei that are located in the central part of basophilic cytoplasm. The origin of the hypertrophic nuclei was suggested to be from myonuclei, not from satellite cells [7,8]. Recent findings, however, have demonstrated the presence of multipotential stem cells in various adult tissues. Adult stem cells isolated from various tissues appear to differentiate into multiple lineages depending on environmental cues [112-116]. Adult muscle-derived stem cells have been shown to differentiate into muscle cell *in vitro *and to contribute to muscle regeneration *in vivo *[105,109,117]. These reports suggest that the muscle derived-stem cells should be further examined as an additional source of the hypertrophic nuclei in infected muscle cell.

As a response of muscle cells to the damage by *Trichinella *larva, de-differentiation occurs. However, the process of muscle cell de-differentiation is not followed by the same process as in muscle cell regeneration after trauma, but results in creating the environment for larva to develop, grow and survive. *Trichinella *larvae grow within the muscle cell at astonishing speed, increasing its volume by about 40% per day [23]. Therefore, this kind development and growth require high consumption of nutrients. The metabolism of protein, glucose and fat in the nurse cell is increased during nurse cell formation [118]. Larvae utilize the de-differentiation of muscle cell to create a suitable environment to nurse it.

#### Collagen capsule

A capsule wall is a prominent non-cellular structure and, as such, one may think it is unique only to *Trichinella *infection, and not shared by the normal muscle cell. An ultrastructural study, however, showed the capsule wall as a sort of simple thickening of the basal lamina that normal muscle cells have. In normal muscles, cellular components, muscle cells and their associated myoblasts (satellite cell) are wrapped together with a single non-cellular sheet, the basal lamina.

The capsule wall has two layers; the inner and outer. The former is produced by the nurse cell and the latter is produced by fibroblasts around the capsule. The spatial relationship among the non-cellular structure and cellular components remains the same before and after capsule formation.

### Parasites utilize cell-biological-systems of hosts to establish parasitism

In this review, the analogy between the processes of nurse cell formation and muscle cell repair has been emphasized. At least the earliest events mobilizing satellite cells seem to be common, but the fate of the proliferated myoblast cell is different. In the former case, the satellite cell differentiates to the muscle cell, but it mis-differentiates to the nurse cell in the latter case. The idea that comes to mind is that *Trichinella*, in order to make its own home, basically utilizes the cell-biological-system of the host which is equipped for the purpose of muscle cell repairing. Since the satellite cell is a progenitor cell located within the capsule wall, a new cell can be continuously supplied from the myoblast, even if the present nurse cell dies. This explains why the nurse cell looks intact and active for years in spite of intracellular parasitism. Thus *Trichinella *can take advantage of the host for its own survival.

How the parasite takes advantage of the host cell biological system to build its home is an interesting issue for parasitologists. Despommier [3] proposed "parakines" as messengers to carry out the communication between parasite and host cells by molecular cross-talking in order to provide life-long coexistence. It was hypothesized that the parakines direct specific cellular behavior by effecting signaling pathways, as cytokines are doing in mammalian host cell.

Thus far, many efforts have been made to identify and characterize the parakines, some of which have provided indirect evidence in support of the hypothesis. Early studies indicated that *Trichinella *antigenic epitopes were detected in the hypertrophy nuclei of infected muscle cell [119,120]. Some nuclear antigens (for example, 79, 86 and 97 kDa proteins) which react with monoclonal antibody to *Trichinella *excretory-secretory (ES) products have been identified and characterized, and their potential effects in regulating nuclear function of the host cell have been studied [121-123]. Vassilatis *et al*. [100] cloned a specific 43 kDa glycoprotein of muscle larva ES which belongs to the basic helix-loop-helix (bHLH) DNA-binding protein family. The bHLH family includes myogenic regulatory factors, suggesting that the 43 kDa ES protein may play a role in the differentiation of host cells. Mak and Ko [124] found a novel DNA-binding protein from ES products, which may function in host genomic reprogramming. Nagano *et al*. [125-127] cloned and characterized several ES proteins, including serine proteinase, serine proteinase inhibitor and Rcd1 (Required cell differentiation 1) – like protein which may involve in host muscle cell differentiation. Tan *et al*. [128] and Wu *et al*. [129] reported that *Trichinella *produces macrophage migration inhibitor (MIF), a cytokine which may protect the parasite from host immune attack.

Though many proteins of *Trichinella *ES products have been cloned and characterized, their precise effects on each step of nurse cell formation (activation, proliferation and re-differentiation of satellite cell, de-differentiation of infected muscle cell) is still unclear. Some of the ES proteins of *Trichinella *are stage specific. Most of the investigated proteins are those produced by late stage of larvae (for example, over 30 days). More attention should be paid to ES products from other *Trichinella *stages. Jasmer and Neary [8] reported that full stichocyte development is not required for host cell cycle re-entry, suggesting that the products of parasitism genes responding to reprogramming host genetic transcription are produced at a very early stage of infection. The proteins shed by the parasite at an early stage of infection seem to be more relevant for determining the mechanism of nurse cell formation.

## Conclusion

The process of nurse cell formation is complex. Many aspects of it are still unknown. The response of infected muscle cell at early stage is quite similar to that occurring in myogensis and muscle regeneration, including the activation, proliferation and differentiation of satellite cell, and cell cycle re-entry. Many genes that play important roles in muscle myogenesis and regeneration are up-regulated and have been proposed as candidate ones involved in nurse cell formation. Some of these genes have been confirmed to be responding to the process of nurse cell development. At the late stage of nurse cell formation, development of infected muscle goes along with the demands of the larva: arrest of cell cycle, the change of basophilic and eosinophilic cytoplasm, involvement of apoptosis and anti-apoptosis and finally transforming into nurse cell. It could be proposed that the process at the beginning is a response of host cells to larval invasion, while the process at a later stage it is reforming or restructuring of host cell processes by larva. Therefore, the present review gives an outline of nurse cell formation, especially on the molecular mechanisms involved.

## Competing interests

The authors declare that they have no competing interests.

## Authors' contributions

All authors engaged in drafting manuscript and approved the final version.

## References

[B1] Maier DM, Zaiman H (1966). The development of lysosomes in rat skeletal muscle in trichinous myositis. J Histochem Cytochem.

[B2] Steward GL, Read CP (1973). Change in RNA in mouse trichinosis. J Parasitol.

[B3] Despommier DD (1998). How does *Trichinella spiralis *make itself at home?. Parasitol Today.

[B4] Wozniak AC, Kong J, Bock E, Pilipowicz O, Anderson JE (2005). Signaling satellite-cell activation in skeletal muscle: markers, models, stretch, and potential alternate pathways. Muscle Nerve.

[B5] Matsuo A, Wu Z, Nagano I, Takahashi Y (2000). Five types of nuclei present in the capsule of *Trichinella spiralis*. Parasitology.

[B6] Wu Z, Matsuo A, Nakada T, Nagano I, Takahashi Y (2001). Different response of satellite cells in the kinetics of myogenic regulatory factors and ultrastructural pathology after *Trichinella spiralis *and *T. pseudospiralis *infection. Parasitology.

[B7] Jasmer DP (1993). *Trichinella spiralis *infected skeletal muscle cells arrest in G_2_/M and cease muscle gene expression. J Cell Biol.

[B8] Jasmer DP, Neary SM (1994). *Trichinella spiralis*: inhibition of muscle larva growth and development is associated with a delay in expression of infected skeletal muscle characteristics. Exp Parasitol.

[B9] Wu Z, Nagano I, Boonmars T, Takahashi Y (2005). A spectrum of functional genes mobilized after *Trichinella spiralis *infection in skeletal muscle. Parasitology.

[B10] Wu Z, Nagano I, Boonmars T, Takahashi Y (2006). Involvement of the c-Ski oncoprotein in cell cycle arrest and transformation during nurse cell formation after Trichinella spiralis infection. Int J Parasitol.

[B11] Wu Z, Nagano I, Takahashi Y (2008). Candidate genes responsible for common and different pathology of infected muscle tissues between *Trichinella spiralis *and *T. pseudospiralis *infection. Parasitol Int.

[B12] Parker MH, Seale P, Rudnicki MA (2003). Looking back to the embryo: defining transcriptional networks in adult myogenesis. Nature Rev Genet.

[B13] Gros J, Manceau M, Thome V, Marcelle CA (2005). Common somitic origin for embryonic muscle progenitors and satellite cells. Nature.

[B14] Relaix F, Rocancourt D, Mansouri A, Buckingham M (2005). A Pax3/Pax7-dependent population of skeletal muscle progenitor cells. Nature.

[B15] Zammit PS, Partridge TA, Yablonka-Reuveni Z (2006). The skeletal muscle satellite cell: the stem cell that came in from the cold. J Histochem Cytochem.

[B16] Tidball JG (1995). Inflammatory cell response to acute muscle injury. Med Sci Sports Exerc.

[B17] Tidball JG (2005). Inflammatory processes in muscle injury and repair. Am J Physiol Regul Integr Comp Physiol.

[B18] Shireman PK (2007). The chemokine system in arteriogenesis and hind limb ischemia. J Vasc Surg.

[B19] Robertson TA, Maley MA, Grounds MD, Papadimitriou JM (1993). The role of macrophages in skeletal muscle regeneration with particular reference to chemotaxis. Exp Cell Res.

[B20] Merly F, Lescaudron L, Rouaud T, Crossin F, Gardahaut MF (1999). Macrophages enhance muscle satellite cell proliferation and delay their differentiation. Muscle Nerve.

[B21] Arnold L, Henry A, Poron F, Baba-Amer Y, van Rooijen N, Plonquet A, Gherardi RK, Chazaud B (2007). Inflammatory monocytes recruited after skeletal muscle injury switch into antiinflammatory macrophages to support myogenesis. J Exp Med.

[B22] Morgan JE, Partridge TA (2003). Muscle satellite cells. Int J Biochem Cell Biol.

[B23] Despommier DD, Aron L, Turgeon L (1975). *Trichinella spiralis*: growth of the intracellular (muscle) larva. Exp Parasitol.

[B24] Boonmars T, Wu Z, Nagano I, Takahashi Y (2004). Expression of apoptosis-related factors in muscles infected with *Trichinella spiralis*. Parasitology.

[B25] Boonmars T, Wu Z, Nagano I, Takahashi Y (2005). *Trichinella pseudospiralis *infection is characterized by more continuous and diffuse myopathy than *T. spiralis *infection. Parasitol Res.

[B26] Boonmars T, Wu Z, Nagano I, Takahashi Y (2005). What is the role of p53 during the cyst formation of *Trichinella spiralis*? A comparable study between knockout mice and wild type mice. Parasitology.

[B27] Wu Z, Nagano I, Boonmars T, Takahashi Y (2005). Tumor necrosis factor receptor-mediated apoptosis in *Trichinella spiralis*-infected muscle cells. Parasitology.

[B28] Despommier DD, Symmans WF, Dell R (1991). Changes in nurse cell nuclei during synchronous infection with *Trichinella spiralis*. J Parasitol.

[B29] Teppema JS, Robinson JE, Ruitenberg EJ (1973). Ultrastructural aspects of capsule formation in *Trichinella spiralis *infection in the rat. Parasitology.

[B30] Baruch AM, Despommier DD (1991). Blood vessels in *Trichinella spiralis *infections: a study using vascular casts. J Parasitol.

[B31] Blotna-Filipiak M, Gabryel P, Gustowska L, Kucharska E, Wranicz MJ (1998). *Trichinella spiralis*: induction of the basophilic transformation of muscle cells by synchronous newborn larvae. II. Electron microscopy study. Parasitol Res.

[B32] Wranicz MJ, Gustowska L, Gabryel P, Kucharska E, Cabaj W (1998). *Trichinella spiralis*: induction of the basophilic transformation of muscle cells by synchronous newborn larvae. Parasitol Res.

[B33] Mancini M, Anderson BO, Caldwell E, Sedghinasab M, Paty PB, Hockenbery DM (1997). Mitochondrial proliferation and paradoxical membrane depolarization during terminal differentiation and apoptosis in a human colon carcinoma cell line. J Cell Biol.

[B34] Desagher S, Martinou JC (2000). Mitochondria as the central control point of apoptosis. Trends Cell Biol.

[B35] Gupta S (2003). Molecular signaling in death receptor and mitochondrial pathways of apoptosis (Review). Int J Oncol.

[B36] Boonmars T, Wu Z, Nagano I, Nakada T, Takahashi Y (2004). Differences and similarities of nurse cells in cysts of *Trichinella spiralis *and *T. pseudospiralis*. J Helminthol.

[B37] Yamochi T, Kaneita Y, Akiyama T, Mori S, Moriyama M (1999). Adenovirus-mediated high expression of BCL-6 in CV-1 cells induces apoptotic cell death accompanied by down-regulation of BCL-2 and BCL-X(L). Oncogene.

[B38] Kojima S, Hatano M, Okada S, Fukuda T, Toyama Y, Yuasa S, Ito H, Tokuhisa T (2001). Testicular germ cell apoptosis in Bcl6-deficient mice. Development.

[B39] Trougakos IP, Gonos ES (2002). Clusterin/apolipoprotein J in human ageing and cancer. Int J Biochem Cell Biol.

[B40] Criswell T, Klokov D, Beman M, Lavik JP, Boothman DA (2003). Repression of IR-inducible clusterin expression by the p53 tumour suppressor protein. Cancer Biol Ther.

[B41] Leskov KS, Klokov DY, Li J, Kinsella TJ, Boothman DA (2003). Synthesis and functional analyses of nuclear clusterin, a cell death protein. J Biol Chem.

[B42] Miyake H, Hara I, Gleave ME, Eto H (2004). Protection of androgendependent human prostate cancer cells from oxidative stress-induced DNA damage by overexpression of clusterin and its modulation by androgen. Prostate.

[B43] Trougakso IP, So A, Jansen B, Gleave ME, Gonos ES (2004). Silencing expression of the clusterin/apolipoprotein J gene in human cancer cells using small interfering RNA induces spontaneous apoptosis, reduced growth ability, and cell sensitization to genotoxic and oxidative stress. Cancer Res.

[B44] Coultas L, Bouillet P, Loveland KL, Meachem S, Perlman H, Adams JM, Strasser A (2005). Concomitant loss of proapoptotic BH3-only Bcl-2 antagonists Bik and Bim arrests spermatogenesis. EMBO J.

[B45] Liu Y, Borchert GL, Surazynski A, Hu CA, Phang JM (2006). Proline oxidase activates both intrinsic and extrinsic pathways for apoptosis: the role of ROS/superoxides, NFAT and MEK/ERK signaling. Oncogene.

[B46] Hu CA, Donald SP, Yu J, Lin WW, Liu Z, Steel G, Obie C, Valle D, Phang JM (2007). Overexpression of proline oxidase induces proline-dependent and mitochondria-mediated apoptosis. Mol Cell Biochem.

[B47] Charge SBP, Rudnicki MA (2004). Celllular and molecular regulation of muscle regeneration. Physiol Rev.

[B48] Zhao P, Hoffman EP (2004). Embryonic myogenesis pathways in muscle regeneration. Dev Dyn.

[B49] Collins CA (2006). Satellite cell self-renewal. Curr Opin Pharmacol.

[B50] Buckingham M (2007). Skeletal muscle progenitor cells and the role of Pax genes. C R Biol.

[B51] Fuchtbauer EM, Westphal H (1992). MyoD and myogenin are coexpressed in regenerating skeletal muscle of the mouse. Dev Dyn.

[B52] Smith CK, Janney MJ, Allen RE (1994). Temporal expression of myogenic regulatory genes during activation, proliferation, and differentiation of rat skeletal muscle satellite cells. J Cell Physiol.

[B53] Cooper RN, Tajbakhsh S, Mouly V, Cossu G, Buckingham M, Butler-Browne GS (1999). In vivo satellite cell activation via Myf5 and MyoD in regenerating mouse skeletal muscle. J Cell Sci.

[B54] Zador E, Bottka S, Wuytack F (2002). Antisense inhibition of myoD expression in regenerating rat soleus muscle is followed by an increase in the mRNA levels of myoD, myf-5 and myogenin and by a retarded regeneration. Biochim Biophys Acta.

[B55] Zammit PS, Heslop L, Hudon V, Rosenblatt JD, Tajbakhsh S, Buckingham ME, Beauchamp JR, Partridge TA (2002). Kinetics of myoblast proliferation show that resident satellite cells are competent to fully regenerate skeletal muscle fibers. Exp Cell Res.

[B56] Yablonka-Reuveni Z, Rivera AJ (1994). Temporal expression of regulatory and structural muscle proteins during myogenesis of satellite cells on isolated adult rat fibers. Dev Biol.

[B57] Walters EH, Stickland NC, Loughna PT (2000). MRF-4 exhibits fiber type- and muscle-specific pattern of expression in postnatal rat muscle. Am J Physiol Regul Integr Comp Physiol.

[B58] Zhou Z, Bornemann A (2002). MRF4 protein expression in regenerating rat muscle. J Muscle Res Cell Motil.

[B59] Kassar-Duchossoy L, Gayraud-Morel B, Gomes D, Rocancourt D, Buckingham M, Shinin V, Tajbakhsh S (2004). Mrf4 determines skeletal muscle identity in Myf5: Myod double-mutant mice. Nature.

[B60] Seale P, Rudnicki MA (2000). A new look at the origin, function, and "stem-cell" status of muscle satellite cells. Dev Biol.

[B61] Hawke TJ, Garry DJ (2001). Myogenic satellite cells: physiology to molecular biology. J Appl Physiol.

[B62] Cornelison DD, Wold BJ (1997). Single-cell analysis of regulatory gene expression in quiescent and activated mouse skeletal muscle satellite cells. Dev Biol.

[B63] Wrobel E, Brzoska E, Moraczewski J (2007). M-cadherin and beta-catenin participate in differentiation of rat satellite cells. Eur J Cell Biol.

[B64] Luo D, Renault VM, Rando TA (2005). The regulation of Notch signaling in muscle stem cell activation and postnatal myogenesis. Semin Cell Dev Biol.

[B65] Hicks C, Johnston SH, diSibio G, Collazo A, Vogt TF, Weinmaster G (2000). Fringe differentially modulates Jagged1 and Delta1 signalling through Notch1 and Notch2. Nat Cell Biol.

[B66] Yang LT, Nichols JT, Yao C, Manilay JO, Robey EA, Weinmaster G (2005). Fringe glycosyltransferases differentially modulate Notch1 proteolysis induced by Delta1 and Jagged1. Mol Biol Cell.

[B67] Carlson ME, Conboy IM (2007). egulating the Notch pathway in embryonic, adult and old stem cells. Curr Opin Pharmacol.

[B68] Berkes CA, Tapscott SJ (2005). MyoD and the transcriptional control of myogenesis. Semin Cell Dev Biol.

[B69] Friday BB, Pavlath GK (2001). A calcineurin- and NFAT-dependent pathway regulates Myf5 gene expression in skeletal muscle reserve cells. J Cell Sci.

[B70] Mourkioti F, Rosenthal N (2005). IGF-1, inflammation and stem cells: interactions during muscle regeneration. Trends Immunol.

[B71] Musaro A, McCullagh K, Paul A, Houghton L, Dobrowolny G, Molinaro M, Barton ER, Sweeney HL, Rosenthal N (2001). Localized Igf-1 transgene expression sustains hypertrophy and regeneration in senescent skeletal muscle. Nat Genet.

[B72] Hill M, Goldspink G (2003). Expression and splicing of the insulin-like growth factor gene in rodent muscle is associated with muscle satellite (stem) cell activation following local tissue damage. J Physiol.

[B73] Hill M, Wernig A, Goldspink G (2003). Muscle satellite (stem) cell activation during local tissue injury and repair. J Anat.

[B74] Bodine SC, Stitt TN, Gonzalez M, Kline WO, Stover GL, Bauerlein R, Zlotchenko E, Scrimgeour A, Lawrence JC, Glass DJ, Yancopoulos GD (2001). Akt/mTOR pathway is a crucial regulator of skeletal muscle hypertrophy and can prevent muscle atrophy in vivo. Nat Cell Biol.

[B75] Rommel C, Bodine SC, Clarke BA, Rossman R, Nunez L, Stitt TN, Yancopoulos GD, Glass DJ (2001). Mediation of IGF-1-induced skeletal myotube hypertrophy by PI(3)K/Akt/mTOR and PI(3)K/Akt/GSK3 pathways. Nature Cell Biol.

[B76] Machida S, Booth FW (2004). Insulin-like growth factor 1 and muscle growth: implication for satellite cell proliferation. Proc Nut Soc.

[B77] Shavlakadze T, Winn N, Rosenthal N, Grounds MD (2005). Reconciling data from transgenic mice that overexpress IGF-I specifically in skeletal muscle. Growth Horm IGF Res.

[B78] Parrizas M, LeRoith D (1997). Insulin-like growth factor-1 inhibition of apoptosis is associated with increased expression of the bcl-xL gene product. Endocrinology.

[B79] Yin D, Tamaki N, Parent AD, Zhang JH (2005). Insulin-like growth factor-I decreased etoposide-induced apoptosis in glioma cells by increasing bcl-2 expression and decreasing CPP32 activity. Neurol Res.

[B80] Morgan DO (1997). Cyclin-dependent kinases: engines, clocks, and microprocessors. Ann Revi Cell Dev Biol.

[B81] Murray AW (2004). Recycling the cell cycle: cyclins revisited. Cell.

[B82] Coqueret O (2002). Linking cyclins to transcriptional control. Gene.

[B83] Ortega S, Malumbres M, Barbacid M (2002). Cyclin D-dependent kinases, INK4 inhibitors and cancer. Biochim Biophys Acta.

[B84] Boonstra J (2003). Progression through the G1-phase of the on-going cell cycle. J Cell Biochem.

[B85] Pines J (1997). Cyclin-dependent kinase inhibitors: the age of crystals. Biochim Biophys Acta.

[B86] Matushansky I, Radparvar F, Skoultchi AI (2000). Manipulating the onset of cell cycle withdrawal in differentiated erythroid cells with cyclin-dependent kinases and inhibitors. Blood.

[B87] Kitzmann M, Fernandez A (2001). Crosstalk between cell cycle regulators and the myogenic factor MyoD in skeletal myoblasts. Cell Mol Life Sci.

[B88] Gartel AL, Radhakrishnan SK (2005). Lost in transcription: p21 repression, mechanisms, and consequences. Cancer Res.

[B89] Bunz F, Dutriaux A, Lengauer C, Waldman T, Zhou S, Brown JP, Sedivy JM, Kinzler KW, Vogelstein B (1998). Requirement for p53 and p21 to sustain G2 arrest after DNA damage. Science.

[B90] Rigberg DA, Blinman TA, Kim FS, Cole MA, McFadden DW (1999). Antisense blockade of p21/WAF1 decreases radiation-induced G2 arrest in esophageal squamous cell carcinoma. J Surg Res.

[B91] De Siervi A, Marinissen M, Diggs J, Wang XF, Pages G, Senderowicz A (2004). Transcriptional activation of p21(waf1/cip1) by alkylphospholipids: role of the mitogen-activated protein kinase pathway in the transactivation of the human p21(waf1/cip1) promoter by Sp1. Cancer Res.

[B92] Shannan B, Seifert M, Boothman DA, Tilgen W, Reichrath J (2006). Clusterin and DNA repair: a new function in cancer for a key player in apoptosis and cell cycle control. J Mol Histol.

[B93] Scaltriti M, Bettuzzi S, Sharrard RM, Caporali A, Caccamo AE, Maitland NJ (2004). Clusterin overexpression in both malignant and nonmalignant prostate epithelial cells induces cell cycle arrest and apoptosis. Br J Cancer.

[B94] Scaltriti M, Santamaria A, Paciucci R, Bettuzzi S (2004). Intracellular clusterin induces G2-M phase arrest and cell death in PC-3 prostate cancer cells. Cancer Res.

[B95] Russell L, Forsdyke DR (1991). A human putative lymphocyte G0/G1 switch gene containing a CpG-rich island encodes a small basic protein with the potential to be phosphorylated. DNA Cell Biol.

[B96] Zandbergen F, Mandard S, Escher P, Tan NS, Patsouris D, Jatkoe T, Rojas-Caro S, Madore S, Wahli W, Tafuri S, Muller M, Kersten S (2005). The G0/G1 switch gene 2 is a novel PPAR target gene. Biochem J.

[B97] Shi Y, Massague J (2003). Mechanisms of TGF-beta signaling from cell membrane to the nucleus. Cell.

[B98] Jasmer DP (1990). *Trichinella spiralis*: altered expression of muscle proteins in trichinosis. Exp Parasitol.

[B99] Jasmer DP, Bohnet S, Prieur DJ (1991). *Trichinella *spp.: differential expression of acid phosphatase and myofibrillar proteins in infected muscle cells. Exp Parasitol.

[B100] Vassilatis DK, Despommier D, Misek DE, Polvere RI, Gold AM, Ploeg LH Van der (1992). Analysis of a 43-kDa glycoprotein from the intracellular parasitic nematode *Trichinella spiralis*. J Biol Chem.

[B101] Carlson MR, Bryant SV, Gardiner DM (1998). Expression of Msx-2 during development, regeneration, and wound healing in axolotl limbs. J Exp Zool.

[B102] Koshiba K, Kuroiwa A, Yamamoto H, Tamura K, Ide H (1998). Expression of Msx genes in regenerating and developing limbs of axolotl. J Exp Zool.

[B103] Odelberg SJ, Kollhoff A, Keating MT (2000). Dedifferentiation of mammalian myotubes induced by msx1. Cell.

[B104] Thompson-Jaeger S, Raghow R (2000). Exogenous expression of Msx1 renders myoblasts refractory to differentiation into myotubes and elicits enhanced biosynthesis of four unique mRNAs. Mol Cell Biochem.

[B105] Grounds MD, White JD, Rosenthal N, Bogoyevitch MA (2002). The role of stem cells in skeletal and cardiac muscle repair. J Histochem Cytochem.

[B106] Hay ED (1959). Microscopic observations of muscle dedifferentiation in regenerating *Amplystoma *limbs. Dev Biol.

[B107] Hay ED, Fischman DA (1961). Origin of the blastema in regenerating limbs of the newt *Triturus viridescens*. Dev Biol.

[B108] McGann CJ, Odelberg SJ, Keating MT (2001). Mammalian myotube dedifferentiation induced by newt regeneration extract. Proc Natl Acad Sci USA.

[B109] Goldring K, Partridge T, Watt D (2002). Muscle stem cells. J Pathol.

[B110] Watt DJ, Jones GE, Goldring K (2004). The involvement of galectin-1 in skeletal muscle determination, differentiation and regeneration. Glycoconj J.

[B111] Chan J, O'Donoghue K, Gavina M, Torrente Y, Kennea N, Mehmet H, Stewart H, Watt DJ, Morgan JE, Fisk NM (2006). Galectin-1 induces skeletal muscle differentiation in human fetal mesenchymal stem cells and increases muscle regeneration. Stem Cells.

[B112] Ferrari G, Cusella-De Angelis G, Coletta M, Paolucci E, Stornaiuolo A, Cossu G, Mavilio F (1998). Muscle regeneration by bone marrow-derived myogenic progenitors. Science.

[B113] Bittner RE, Schofer C, Weipoltshammer K, Ivanova S, Streubel B, Hauser E, Freilinger M, Hoger H, Elbe-Burger A, Wachtler F (1999). Recruitment of bone-marrow-derived cells by skeletal and cardiac muscle in adult dystrophic mdx mice. Anat Embryol.

[B114] Jankowski RJ, Deasy BM, Cao B, Gates C, Huard J (2002). The role of CD34 expression and cellular fusion in the regeneration capacity of myogenic progenitor cells. J Cell Sci.

[B115] LaBarge MA, Blau HM (2002). Biological progression from adult bone marrow to mononucleate muscle stem cell to multinucleate muscle fiber in response to injury. Cell.

[B116] Qu-Petersen Z, Deasy B, Jankowski R, Ikezawa M, Cummins J, Pruchnic R, Mytinger J, Cao B, Gates C, Wernig A, Huard J (2002). Identification of a novel population of muscle stem cells in mice: potential for muscle regeneration. J Cell Biol.

[B117] Seale P, Sabourin LA, Girgis-Gabardo A, Mansouri A, Gruss P, Rudnicki MA (2000). Pax7 is required for the specification of myogenic satellite cells. Cell.

[B118] Steward GL, Campbell WC (1983). Pathophysiology of the muscle phase. Trichinella and Trichinosis.

[B119] Despommier DD, Gold AM, Buck SW, Capo V, Silberstein D (1990). *Trichinella spiralis*: secreted antigen of the infective L1 larva localizes to the cytoplasm and nucleoplasm of infected host cells. Exp Parasitol.

[B120] Lee DL, Ko RC, Yi XY, Yeung MH (1991). *Trichinella spiralis*: antigenic epitopes from the stichocytes detected in the hypertrophic nuclei and cytoplasm of the parasitized muscle fibre (nurse cell) of the host. Parasitology.

[B121] Yao C, Bohnet S, Jasmer DP (1998). Host nuclear abnormalities and depletion of nuclear antigens induced in *Trichinella spiralis*-infected muscle cells by the anthelmintic mebendazole. Mol Biochem Parasitol.

[B122] Yao C, Jasmer DP (1998). Nuclear antigens in *Trichinella spiralis *infected muscle cells: nuclear extraction, compartmentalization and complex formation. Mol Biochem Parasitol.

[B123] Yao C, Jasmer DP (2001). *Trichinella spiralis*-infected muscle cells: abundant RNA polymerase II in nuclear speckle domains colocalizes with nuclear antigens. Infect Immun.

[B124] Mak CH, Ko RC (2001). DNA-binding activity in the excretory-secretory products of *Trichinella pseudospiralis *(Nematoda: Trichinelloidea). Parasitology.

[B125] Nagano I, Wu Z, Nakada T, Boonmars T, Takahashi Y (2003). Molecular cloning and characterization of a serine proteinase gene of *Trichinella spiralis*. J Parasitol.

[B126] Nagano I, Wu Z, Nakada T, Matsuo A, Takahashi Y (2001). Molecular cloning and characterization of a serine proteinase inhibitor from *Trichinella spiralis*. Parasitology.

[B127] Nagano I, Wu Z, Takahashi Y (2006). Molecular cloning and characterization of an Rcd1-like protein in excretory-secretory products of *Trichinella pseudospiralis*. Parasitology.

[B128] Tan TH, Edgerton SA, Kumari R, McAlister MS, Roe SM, Nagl S, Pearl LH, Selkirk ME, Bianco AE, Totty NF, Engwerda C, Gray CA, Meyer DJ (2001). Macrophage migration inhibitory factor of the parasitic nematode *Trichinella spiralis*. Biochim J.

[B129] Wu Z, Boonmars T, Nagano I, Nakada T, Takahashi Y (2003). Molecular expression and characterization of a homologue of host cytokine macrophage migration inhibitory factor from *Trichinella *spp. J Parasitol.

